# Effects of spousal migration on access to healthcare for women left behind: A cross-sectional follow-up study

**DOI:** 10.1371/journal.pone.0260219

**Published:** 2021-12-02

**Authors:** Heidi S. West, Mary E. Robbins, Corrina Moucheraud, Abdur Razzaque, Randall Kuhn

**Affiliations:** 1 Department of Health Policy and Management, Fielding School of Public Health, University of California Los Angeles, Los Angeles, CA, United States of America; 2 Department of Gender Studies, University of California Los Angeles, Los Angeles, CA, United States of America; 3 Health and Population Surveillance Division, ICDDR, B, Dhaka, Bangladesh; 4 Department of Community Health Sciences, Fielding School of Public Health, University of California Los Angeles, Los Angeles, CA, United States of America; Tulane University School of Public Health and Tropical Medicine, UNITED STATES

## Abstract

**Background:**

Women left behind by migration represent a unique and growing population yet remain understudied as key players in the context of migration and development. Using a unique longitudinal survey of life in Bangladesh, the Matlab Health and Socioeconomic Surveys, we examined the role of spousal migration in healthcare utilization for women. The objective of this study was to assess realized access to care (do women actually get healthcare when it is needed) and consider specific macrostructural, predisposing, and resource barriers to care that are related to migration.

**Methods and findings:**

In a sample of 3,187 currently married women, we estimated multivariate logistic and multinomial regression models controlling for a wide range of baseline sociodemographic factors measured as far back as 1982. Our analyses also controlled for selection effects and explored two mechanisms through which spousal migration can affect healthcare utilization for women, remittances and frequent contact with spouses. We found that women with migrant spouses were approximately half as likely to lack needed healthcare compared to women whose spouses remained in Bangladesh (predicted probability of not getting needed healthcare 11.7% vs. 21.8%, p<0.001). The improvements in access (logistic regression coefficient for lacking care for left-behind women -0.761 p<0.01) primarily occurred through a reduction in financial barriers to care for women whose spouses were abroad.

**Conclusions:**

Wives of international migrants showed significantly better access to healthcare even when accounting for selection into a migrant family. While the overall story is one of positive migration effects on healthcare access due to reductions in financial barriers to care, results also showed an increase in family-related barriers such as not being permitted to get care by a family member or travel alone to a facility, indicating that some of the benefits of migration for women left behind may be diluted by gendered family structures.

## Introduction

Women left behind by migration represent a unique and growing population yet remain understudied as key players in the context of migration and development. Up to one-third of the world’s international migrants are on temporary work visas, most of whom are men with families left behind [1]. In 2017, over 1 million Bangladeshis migrated overseas for work, with men accounting for almost 90% of this group [2]. The mechanisms through which migration impacts national economic development garner much attention, with migrant remittances totaling more than three times all development aid [3–7]. How do these macro level shifts in labor and economic resources play out in the lives of left-behind families? We considered this question by following a large sample of women left behind in a high out-migration context in rural Bangladesh.

Evidence on the health impacts of migration on families left behind remains limited, particularly with respect to spouses [8]. Most studies on migrant sending communities (communities of origin for migrants) are geographically focused on China and Mexico and look at children and parents of migrants. The migration and health literature highlights both the positive and negative effects of migrating parents on psychosocial, educational, and physical health outcomes for children. [9–15]. For older adults left behind we see positive effects on nutrition, morbidity, and mortality [16–18]. However, studies of spousal health impacts remain limited and focus primarily on HIV or other sexual and reproductive health issues [19–22].

Understanding how migration relates to determinants of healthcare utilization in sending communities can help health systems improve access, disease treatment and prevention, and target scarce resources. For women in prime adulthood who experience relatively few measurable morbidities, access to health care may be an important driver of long-term health. The objective of this study was to assess realized access to care (do women actually get healthcare when it is needed) and consider specific macrostructural, predisposing, and resource barriers to care, drawing on the influential Andersen model for explaining healthcare utilization [23–25]. We address these mechanisms in the context of the pluralistic Bangladeshi health system characterized by widely available yet variable quality healthcare [26].

One way in which migration may affect health is through remittances, which increase household income and reduce resource barriers to care -seeking among the left-behind. The literature on remittances and how they affect healthcare decision-making, mobility, and access for the left-behind is inconclusive, showing increased access to some services and not others, and improvements in decision-making and autonomy are more dependent on the specific timing and patterns of remittances than the amount received [27–30]. Remittances, often used for basic consumption needs, [18, 31] also change labor force participation in different ways for men and women, leading to shifts in responsibility and time constraints that impact women’s ability to seek and afford healthcare services [32–34]. In some cases, the absence of men leads to increased responsibility among women, as they are expected to take on their own tasks and the tasks of their husband [35].

A husband’s migration has substantial implications for women’s health and autonomy, both through the remittances they send home and also the constraints induced by spousal absence and adjustments in household headship [28, 36–39]. The measurable, economic-driven effects must be understood in the context of gendered political and social constraints on women’s residential patterns and control over resources [36]. For example, women in Bangladesh were legally barred from migrating for work until 2007, and the common practice of *purdah*, a set of norms that promote the seclusion of women, determine opportunities for women and their roles within a household [2, 40, 41]. Studies from Egypt [42], Mexico [43], Armenia and Guatemala [44], and Nepal [38] have reported that with male spousal migration, women gain household and financial decision-making power. On the other hand, men have also been shown to retain primary control within the household as they still maintain control over household resources and dynamics [42]. The effects of migration may also be mediated by factors associated with gendered social and economic relationships underlying the migration experience. As is common in Bangladesh, the absence of a spouse may lead to reconfiguration in living situations and household dynamics, making natal family members and in-laws cross-generational influencers for their daughters or daughters-in-law in areas of health and reproductive agency [45–48]. In some cases, mothers-in-law may contribute to poor treatment, disempowerment, and health of young married women through reinforcement of inequitable gender dynamics and roles in the home [37, 49–51]. Similarly, the extent of spousal contact with the absent husband may mediate the level of autonomy in either direction. Higher levels of spousal contact may reflect a stronger and more supportive spousal relationship, but it may also indicate an absent spouse who maintains tight control over a woman’s mobility and healthcare decision-making.

## Methods

### Study design

This observational study compared healthcare utilization, specific barriers to healthcare access, and health outcomes for women in terms of their exposure to spousal migration. Taking advantage of the data characteristics, specifically the large sample size, high percentage of migrants, and the availability of longitudinal data, we estimated differences in healthcare utilization and health outcomes attributable to migration with intergenerational controls for selection into migration, buoyed by significant sampling power. We also considered the mediating mechanisms of remittances and social contact between spouses.

The treatment, spousal migration, was not randomly assigned. Factors that can increase migration propensity such as available household assets, more education, existence of networks, and the health of spouses may lead to an overstatement of the positive effect of spousal migration on women’s healthcare utilization. Although a panel design can address some of these concerns by observing healthcare utilization after controlling for given baseline assets, education and health status concerns still persist regarding the latent aspects of self-selection that are too difficult to control for in statistical models [52]. Accordingly, we also estimated 2-stage propensity score models in which baseline variables were used to predict selection into international spousal migration.

### Data source

Data were drawn from three sources. Since 1974, the International Center for Diarrheal Disease Research, Bangladesh (icddr,b) has implemented a Health and Demographic Surveillance System (HDSS) in Matlab, Bangladesh, collecting data on nearly the entire population of 200,000 people. Migration histories for families were drawn from the HDSS 1982–2014. In 1996, the widely-utilized Matlab Health and Socioeconomic Survey (MHSS1) was conducted on a representative sample of 7% of households, followed by MHSS2 in 2012–2014 (10,434 households) which had unusually successful migrant tracking, capturing 92–94% of surviving members of all key age-sex cohorts regardless of whether they had migrated out of the Matlab area [53, 54]. MHSS1 1996 survey data were employed for generational controls of household assets, an important socioeconomic measure, and for sensitivity analyses to compare healthcare access for women whose husbands had varying migration status between the two survey waves. Outcome measures and remaining covariates are from MHSS2 2012–2014. Analysis was conducted on a sample of 3,187 currently married women between the ages of 15–45 who were part of an MHSS1 household in 1996 and completed the full adult survey in 2012. Given high rates of out-migration among prime age adults, each respondent was administered a *Non-Coresident Spouse* module, which collected data on the location of non-household spouses and the extent of financial transfers and phone/email contact. Protection of human participants during fieldwork and data analysis was ensured under icddr,b Ethical Review Committee Protocol #PR-10005, "Long-term effects of health and development interventions in rural Bangladesh." Written informed consent was obtained from participants.

### Dependent variables

We examined outcomes across three domains all measured at MHSS2 (2012–2014): access to needed healthcare, barriers to healthcare utilization, and self-reported health. The main outcome measure in this study was access to healthcare. Respondents were asked “When was the last time that you needed health care from a health professional like a doctor or nurse?” If the response was less than 3 years ago then they were asked the survey question "Have you ever needed health care in the past 3 years but did not get it?" with a yes answer indicating that the respondent was prevented from utilizing needed healthcare. This binary measure of whether or not someone was lacking needed healthcare excludes people who did not need healthcare.

For those who could not get needed healthcare, a follow-up question measured the barriers to utilizing needed care. The responses were collapsed into four categories: i) financial reasons, ii) responsibility reasons–busy/couldn’t get time off, iii) family reasons–family would not permit, required to have escort and no one to accompany, and iv) other reasons–transportation, too ill to travel, did not know where to go, other individual reasons.

Self-reported health status, a three-category variable (poor health/unhealthy, fairly healthy, good health/healthy) was operationalized into a binary outcome variable and multiple specifications were tested. The results reported in Appendix E in S1 Appendix show two specifications of the binary variable: poor health versus fair health or healthy, and poor or fair health versus healthy. While the specific health status outcomes are not the principal focus of this paper, self-reported health is a reliable measure of whether or not an individual would perceive that they needed healthcare [55], which was a key component of our main outcome measure. When self-reported health was used as an independent variable in healthcare access models, we used the full 3 category specification (healthy, fairly healthy, poor health/unhealthy).

### Independent variables

The primary independent variable was a binary measure of the husband’s migration status (international migrant, not an international migrant) at the time of MHSS2 (2012–2014). Multiple specifications of the independent variable were tested including a categorical version that captured the current location of the husband regardless of migration status and dummy versions of each of these categories: co-residents, domestic migrants, international migrants, and husbands who were living outside of the household but not for migration reasons (separation, only recently married and haven’t joined households, living with another wife’s family in cases of polygamy). Given our focus on international migration and the limited association of other forms of non-coresidence on healthcare utilization, the presentation of the main results focused exclusively on the binary international migrant versus not international migrant variable. In sensitivity analyses we also tested how the timing of spousal migration in relation to healthcare utilization may have impacted results (Appendix A in S1 Appendix). To do this we combined data on spousal migration status from the 1996 and 2012–14 rounds of the survey to create variables that indicated whether or not a woman stably had a non-migrant spouse, stably had a migrant spouse, had a migrant spouse at MHSS1 only, or had a migrant spouse at MHSS2 only. Women who were not married at MHSS1 were categorized based on the migration status of their spouse at MHSS2.

Mediation analyses looked at the enabling or resource-based characteristics of remittances (any remittance, remittance value) and frequency of spousal contact, both measured in MHSS2 (2012–14). Remittance variables were derived from the question, asked of women with non-coresident spouses only, “Did you receive money, loan repayment, tuition, health care costs (including treatment) from [spouse name] in the past 12 months? If yes, how much?” Binary versions indicated whether or not the respondent had received any remittances. Continuous versions were reported in Taka and converted to US Dollars. A square root transformation was performed to address issues of non-normality of the distribution. Spousal contact variables were measured from the question “How often were in contact through telephone, text messages, e-mail or post contact with [non-coresident spouse name] in the past 12 months?” Responses included never, at least once a year, at least once a month, at least once a week, everyday. Most women were in contact with their non-coresident spouses everyday so this variable was dichotomized to “everyday” “not everyday.” In primary analyses, women with coresident spouses were coded as “everyday.” Sensitivity analyses were conducted on the subsample of women with non-coresident spouses only.

We also controlled for the various pre-disposing, enabling and need characteristics of age, education, minor children, rural versus urban location, household headship, self-reported health, father’s education (all measured in 2012–14), father’s and brother’s migration history (measured 1982–2014), and household assets (measured in 1996). Age and education were measured as ordinal variables in 5-year increments. Minor children were measured as a dichotomous variable indicating whether or not there were children under 18 years of age in the home. Specifications indicating the number and sex of the children were also tested. In addition to controlling for covariates that are associated with healthcare need and access, we included measures that predicted whether or not a woman would have a migrant husband (family migration history) and tested for interactions and added variables in the model to account for the moderation effects of the independent variables with each other and the outcome. It is important to control extensively for father’s and brother’s migration history because as demonstrated in theories of cumulative causation [56] family migration is a significant confounder and predictor of selection into a relationship with a migration spouse. Both father’s and brother’s migration were measured as binary variables indicating whether or not they were a migrant during the period 1982–2014. Women without brothers were coded as not having a migrant brother. If women had multiple brothers, migration by one or more was coded as having a migrant brother regardless of the status of other brothers. In order to better isolate the effect of spousal migration instead of family migration generally, we include these controls.

### Analyses

Statistical analyses were performed using Stata Statistical Software: Release 15, College Station, TX: StataCorp LLC. For testing overall differences across categories within variables, chi-square tests and t-tests, and were used to calculate p values. Statistical estimates of healthcare utilization, barriers to access and self-reported health were based on weighted logistic (for binomial variables) and weighted multinomial (for categorical variables) regression models controlling for time-invariant individual characteristics (age, education), presence of minor children, geographic location (rural/urban), household structure (relationship to household head), remittances, social contact between spouses, parental schooling, father’s and brother’s migration history and household assets. Survey weights were used to account for the complex survey design and selection of baris (patrilineally-related clusters of households), households, and individuals in both MHSS1 and MHSS2. Variables that were included in the final model were selected using a combination of a review of the literature, the model fit statistics, and formal tests on individual predictors and interaction terms.

Following the framework developed by Rosenbaum and Rubin and its recent application in migration related studies, we tested multiple selection control models [16, 27, 57–60]. Our propensity score analysis focused on predisposing variables well-known to impinge on the migration decision, including household socioeconomic status, family migration history, and education. For each outcome, we tested models that did not include propensity score adjustments, models that restricted the sample to the area of common support (ACS), models that used the restricted sample and a linear propensity score term as a control, and models that used a restricted sample and controls for propensity block. The second analytic approach that we employed was the calculation of the average treatment effect based on propensity score matching.

## Results

### Descriptive analysis

Table 1 provides background on the characteristics of the women in the sample including factors that differentiate migrant families. Seventeen-and-a-half percent of married women had a husband who was living abroad at the time of survey. Women with international migrant spouses differed from women who lived together with their husbands or whose husbands were domestic migrants across a range of sociodemographic factors. Women with international migrant spouses were younger on average (29 years old compared to 31 years old) and had more education (87% > 4 years compared to 69% > 4 years of education). Eighty-nine percent of all respondents had minor children at home (mean number of minor children was 1.7), only 86% of women with migrant spouses had minor children. Additionally, women with migrant spouses had greater household assets (mean $7853.00) compared to women without international migrant spouses (mean $6027.00).

**Table 1:** Characteristics of respondents, by spousal migration status.

	Spouse is Not International Migrant *n =* 2629	International Migrant Spouse *n =* 558	Total *n =* 3187
Mean Age***	31.2 (7.5)	29.1 (6.9)	30.8 (7.4)
Has minor children at home* (mean #)	89.3% (1.8)	86.4% (1.6)	88.8% (1.7)
Lives in urban area***	26.2%	10.8%	23.5%
Received any remittances (last 12 mos)***	13.1%	90.5%	26.7%
Remittances received (USD, last 12 mos)***	$151.37 ($926.85)	$2515.53 ($3475.32)	$564.82 ($1903.84)
Everyday contact with spouse***	96%	68.2%	91.2%
Respondent relationship to household head ***			
Nuclear: HH Head/Wife of HH head	79.4%	51.7%	74.5%
Multigen: Head is Bio/Natal family	3.2%	11.7%	4.6%
Multigen: Head is In-Law	17.5%	36.6%	20.8%
Respondent’s Education***			
0 years	11.6%	2.9%	10.1%
1–4 years	19.1%	10.2%	17.5%
5–9 years	50.7%	61.8%	52.7%
10+ years	18.6%	25.1%	19.7%
^a^ Father was international migrant***	5.8%	13.8%	7.2%
^a^ Brother was international migrant***	26.0%	41.8%	28.7%
^b^ Household Assets (USD)	$6026.57 ($11428.59)	$7852.72 ($10366.23)	$6345 ($11269.86)

Data are given as mean (SD) or percent.

*** p<0.001

** p<0.01

* p<0.05, p values indicate significance of Chi-square or T-test statistics on difference between women with and without international migrant spouses.

Source: MHSS2 (2012–2014) except where noted.

^a^ Matlab Health and Demographic Surveillance System 1982–2014, any brother.

^b^ MHSS1 1996–1997 for wife’s household (sum value of assets across all productive and non-productive types).

Migration-related characteristics also differed between the groups, with significantly higher percentages of women with migrant spouses also having migrant fathers and brothers. While 13% of women with domestic migrant spouses received remittances, over 90% of women with international migrant spouses received remittances and the average annual amount was almost 17 times greater, $2516.00 compared to $151.00. More than twice as many women without international migrant spouses lived in an urban area compared to women with international migrant spouses. Women with international migrant spouses also experienced different family arrangements, being much more likely to have a biological family member (11.7% versus 3.2%) or in-law as household head (36.6% versus 17.5%) and less likely to be a head or spouse of head of household (51.7% versus 79.4%).

Women with migrant spouses saw improved access to healthcare: women with international migrant spouses (9.1%) were half as likely to lack needed healthcare compared to other married women (20.6%) (Table 2). The specific barriers to healthcare access varied for women depending on the migration status of their spouse. Seventy-percent fewer women with international migrant spouses experienced financial barriers to care, the most significant utilization barrier for all groups. While the number of people experiencing other barriers was small, the difference in the proportions of women experiencing them were significantly different based on the migration status of their spouse. Women with international migrant spouses experienced a 40% increase in family related barriers overall (0.5% vs 0.7%). There were no significant differences in self-reported health between women with and without migrant spouses.

**Table 2:** Description of respondents healthcare access by spousal migration status.

	Spouse is Not International Migrant *n =* 2629	International Migrant Spouse *n =* 558	Total n = 3187
Healthy	56.2%	59%	56.7%
Fairly Healthy	33.1%	33.0%	33.0%
Unhealthy/poor health	10.7%	8.1%	10.3%
Unable to access needed healthcare***	20.6%	9.1%	18.6%
Healthcare Utilization Barriers ***			
No access issues	79.2%	90.8%	81.3%
^a^Reason for not accessing healthcare			
1. Financial, couldn’t afford	17.3% (83.2%)	5.2% (56.9%)	15.2% (80.9%)
2. Busy, no time off wk	1.3% (6.4%)	1.3% (13.7%)	1.3% (7%)
3.Family wouldn’t allow /Couldn’t go alone	0.5% (2.2%)	0.7% (7.8%)	0.5% (2.7%)
4. Other reason	1.7% (8.2%)	2% (21.6%)	1.8% (9.4%)

Source: MHSS2 (2012–2014).

^a^Percent of total sample (percent of those who experienced a barrier that prevented access to care).

*** p<0.001, ** p<0.01, * p<0.05.

p values indicate significance of Chi-square test on difference between women with and without international migrant spouses.

### Multivariate analysis

#### Healthcare utilization

International spousal migration remained a significant predictor of women’s ability to access healthcare in multivariate models controlling for family structure, self-reported health, age, minor children, individual and father’s education, rural/urban location, household assets, and family migration history. Having a migrant spouse reduced the probability that a woman would not get needed healthcare (logistic regression coefficient: -0.786, p<0.001) (Table 3, Model 3).

**Table 3:** Multivariate analysis of characteristics associated with not accessing needed healthcare.

	1. Demo	2. SRH	3. Full	4. Remit	5. Sp Contact
Has International Migrant Spouse	-0.638**	-0.683**	-0.786***	-0.642*	-0.761**
	(0.234)	(0.227)	(0.237)	(0.272)	(0.293)
Age (ref = 15–19 years)					
20–24	0.400	0.241	0.272	0.273	0.287
	(0.506)	(0.525)	(0.480)	(0.480)	(0.491)
25–29	0.589	0.315	0.415	0.412	0.446
	(0.498)	(0.509)	(0.461)	(0.460)	(0.473)
30–34	0.824	0.545	0.761	0.759	0.812
	(0.504)	(0.517)	(0.468)	(0.468)	(0.481)
35–39	1.065*	0.585	0.740	0.729	0.761
	(0.507)	(0.516)	(0.475)	(0.475)	(0.486)
40–44	1.068*	0.611	0.745	0.732	0.782
	(0.503)	(0.514)	(0.471)	(0.472)	(0.484)
Respondent’s education (ref = none)					
1–4 years	-0.0752	-0.149	-0.0429	-0.0404	-0.0409
	(0.214)	(0.215)	(0.224)	(0.224)	(0.224)
5–9 years	-0.405*	-0.549**	-0.323	-0.310	-0.282
	(0.205)	(0.213)	(0.237)	(0.237)	(0.237)
10+ years	-1.694***	-1.763***	-1.394***	-1.385***	-1.352***
	(0.268)	(0.274)	(0.321)	(0.321)	(0.321)
Has minor children at home	0.702**	0.770**	0.684*	0.674*	0.670*
	(0.253)	(0.269)	(0.272)	(0.273)	(0.274)
Self-reported health (ref = healthy)					
Fairly healthy		0.915***	0.933***	0.935***	0.928***
		(0.144)	(0.146)	(0.146)	(0.146)
Unhealthy/poor health		1.716***	1.650***	1.644***	1.647***
		(0.192)	(0.196)	(0.196)	(0.199)
Family Structure (ref: nuclear- head/wife of head)					
Multigen: Head is Bio/Natal			0.210	0.274	0.261
			(0.324)	(0.330)	(0.343)
Multigen: Head is In-Law			0.0552	0.0667	0.0704
			(0.198)	(0.198)	(0.198)
Lives in urban area			-1.163***	-1.179***	-1.176***
			(0.209)	(0.212)	(0.212)
^a^Household Assets (log)			-0.152**	-0.152**	-0.150**
			(0.0534)	(0.0533)	(0.0532)
Father’s Education (ref = none)					
1–4 years			-0.0459	-0.0534	-0.0627
			(0.202)	(0.203)	(0.204)
5–9 years			-0.191	-0.193	-0.194
			(0.183)	(0.183)	(0.183)
10+ years			0.0285	0.0303	0.0541
			(0.246)	(0.246)	(0.247)
^b^Father was international migrant			0.506	0.507	0.529
			(0.303)	(0.305)	(0.310)
^b^Brother was international migrant			-0.140	-0.149	-0.162
			(0.164)	(0.164)	(0.165)
Received Remittances				-0.195	-0.378
				(0.208)	(0.208)
Everyday contact or spouse is co-resident					-0.659*
					(0.285)
Observations	3,187	3,187	3,187	3,187	3,187

Source: MHSS2 (2012–2014) except where noted

*** p<0.001

** p<0.01

* p<0.05, Logistic Regression coefficients with robust standard errors in parentheses

^a^ MHSS1 1996–1997 for wife’s household (sum value of assets across all productive and non- productive types)

^b^ Matlab Health and Demographic Surveillance System 1982–2014, any brother.

Other factors that were associated with healthcare utilization included age, education, having minor children at home, household assets, poorer health, and urban location (Table 3). Older women were more likely to experience barriers that prevented them from accessing care compared to younger women, but age only became significant at 35 years and above and only in the demographics model (Model 1).

Women with more education were less likely to experience barriers to care compared to less educated women. Having minor children at home increased the likelihood that women would experience barriers to accessing needed healthcare and this statistically significant effect held across all models. Having greater household assets in 1996 was also a significant predictor for enhanced ability to obtain needed healthcare in 2012. Not surprisingly, women who had poorer health also experienced worse access to healthcare. Poor health predicts greater need, meaning more financial output and mobility are needed, both of which can be limited by illness and disability. While self-reported health status was significantly associated with more barriers to care, it did not change the relationship between spousal migration and healthcare utilization for women as we see from examining models 1, 2, and 3. Women who lived in an urban area were less likely to experience barriers that prevented them from accessing care compared to women who lived in a rural area (coefficient: -1.163, p<0.001). Regardless of the fact that the effect of living in an urban area was quite strong and held across all bivariate and multivariate tests, and women whose spouses remained in Bangladesh were more likely to live in urban areas (26.2% compared to 10.8% of women with international migrant spouses), international migration still had a significant effect in reducing barriers to healthcare access for married women. The effects of these covariates hold across all models for the main outcome.

#### Analysis of mechanisms

There are multiple mechanisms through which migration can positively or negatively influence healthcare utilization for women left behind. We tested mediation models that included different specifications of remittances received and frequency of social contact between spouses (Table 3, Appendices B, C in S1 Appendix). When adding remittances into the models (Table 3 Model 4), we saw that some of the effects of migration were a result of remittances received as the magnitude and significance of the migration variable were reduced. However, the coefficients for remittances were small and not statistically significant (Table 3 Models 4 & 5, Appendix B in S1 Appendix). When controlling for the mediation effects of social contact (everyday in-person contact for those with co-resident spouses or phone/video/ chat for those with migrant spouses) and remittances received (Table 3 Model 5), the effect of migration was only marginally reduced (coefficient: -0.761, p<0.01) and everyday social contact had a similar positive effect on healthcare access as the overall international migration variable (coefficient: -0.659, p<0.05).

#### Treatment selection and other sensitivity analyses

Because the 2-stage propensity score approach did not alter the results, we reported only the more intuitive single-stage estimates in the main tables. Appendix D in S1 Appendix provides estimates for main effects and covariates for different specifications of the propensity models which were nearly identical to the specifications without propensity score adjustments. The area of common support (ACS) included propensities between 0.012 and 0.744, accounting for all but 7 observations from the full sample (0.0025%). There were 8 blocks, and the balancing property is satisfied. Using propensity scores to control for selection into the treatment of having a migrant spouse, we found the effect of migration on women’s healthcare utilization was robust to a variety of propensity adjustments: 1) restricting analysis to the area of common support, 2) controlling for propensity score, 3) controlling for propensity block, and 4) estimating the average treatment effect. The average treatment effect (ATE) of having an international migrant spouse in the full model without mediation was -0.102, p<0.001, meaning that spousal migration reduced the average probability of lacking needed healthcare by about half (ATET: -0.10, p<0.001). Mediation models with controls for the receipt of remittances and social contact with their spouse had slightly smaller and less significant migration effects (ATE: -0.087, ATET: -0.108, p<0.001).

We conducted multiple sensitivity analyses in addition to addressing the selection effects as outlined above. These included 1) alternate specifications of the primary regressor to test for the effects of duration between exposure to spousal migration and healthcare access (Appendix A in S1 Appendix); 2) disaggregating women with spouses in Bangladesh by spouse’s location (out of district, out of household, same household) (results not shown); 3) alternate specifications of the remittances variable (Appendix B in S1 Appendix); and 4) restricting analysis only to women with non-coresident spouse (Appendix C in S1 Appendix). None of these choices affected the results.

#### Barriers to care

In multivariate multinomial and logistic regression models, having an international migrant spouse is associated with a decrease in financial barriers to care (coefficient: -1.037, p<0.01) and an increase in family related barriers to healthcare utilization (coefficient: 1.702, p<0.05) (Table 4). Spousal migration was associated with reductions in barriers related to responsibility (busy/can’t get time off) and other barriers, but these were not statistically significant.

**Table 4:** Multinomial analysis of barriers to accessing needed healthcare.

	1. Financial	2. Busy	3. Family	4. Other
Has International Migrant Spouse	-1.037**	-0.423	1.702*	-1.057
	(0.355)	(0.707)	(0.691)	(0.667)
Age (ref = 15–19 years)				
20–24	0.660	-0.0305	0.940	0.128
	(0.738)	(0.829)	(1.365)	(0.974)
25–29	0.781	-0.106	0.411	0.863
	(0.716)	(0.786)	(1.110)	(1.116)
30–34	1.213	0.0167	1.311	0.680
	(0.724)	(0.803)	(1.147)	(1.117)
35–39	1.200	-0.0631	-0.439	1.081
	(0.724)	(0.966)	(1.264)	(1.189)
40–44	1.207	-0.764	-0.605	1.033
	(0.720)	(1.202)	(1.417)	(1.161)
Respondent’s education (ref = none)				
1–4 years	0.0450	0.889	-0.201	-0.281
	(0.234)	(1.160)	(1.441)	(0.659)
5–9 years	-0.216	1.106	0.0693	-0.423
	(0.254)	(1.169)	(1.041)	(0.615)
10+ years	-1.646***	0.654	-16.70***	-0.598
	(0.390)	(1.275)	(1.069)	(0.768)
Has minor children at home	1.034**	0.226	0.579	-0.172
	(0.316)	(0.773)	(1.154)	(0.699)
Self-reported health (ref = healthy)				
Fairly healthy	1.043***	0.0885	1.436*	0.377
	(0.165)	(0.447)	(0.707)	(0.365)
Unhealthy/poor health	1.743***	0.781	2.914***	0.325
	(0.212)	(0.590)	(0.666)	(0.523)
Family Structure (ref: nuclear- head/wife of head)				
Multigen: Head is Bio/Natal	-0.161	1.740*	-0.127	-0.164
	(0.449)	(0.719)	(1.603)	(0.720)
Multigen: Head is In-Law	-0.168	0.927	-0.602	0.911*
	(0.232)	(0.514)	(0.818)	(0.463)
Lives in urban area	-1.170***	-0.811	0.0726	-2.482***
	(0.238)	(0.593)	(0.845)	(0.592)
^a^Household Assets (log)	-0.174**	-0.108	0.268	-0.131
	(0.0588)	(0.122)	(0.164)	(0.172)
Father’s Education (ref = none)				
1–4 years	-0.312	-0.625	0.995	1.335**
	(0.225)	(0.632)	(0.740)	(0.470)
5–9 years	-0.281	0.122	0.458	0.249
	(0.207)	(0.481)	(0.759)	(0.454)
10+ years	-0.313	1.008	1.297	0.618
	(0.292)	(0.533)	(0.861)	(0.574)
^b^Father was international migrant	0.638	0.884	-2.271	-1.134
	(0.359)	(0.580)	(1.278)	(0.759)
^b^Brother was international migrant	-0.194	-0.908*	-0.340	0.536
	(0.184)	(0.457)	(0.615)	(0.380)
Household Received Remittances	-0.598*	-0.285	-0.127	0.261
	(0.238)	(0.648)	(0.562)	(0.545)
Everyday contact or spouse is co-resident	-1.027**	1.820*	1.022	-0.686
	(0.338)	(0.708)	(1.146)	(0.659)
Observations	3,187	3,187	3,187	3,187

Source: MHSS2 (2012–2014) except where noted

*** p<0.001

** p<0.01

* p<0.05, Multinomial Regression coefficients, robust standard errors in parentheses. Reference group: able to access all needed healthcare in last 3 years (no access issues), Other: transportation, too ill, didn’t know where to go (all <1% of responses), & "other specify," ^a^MHSS1 1996–1997 for wife’s household (sum value of assets across all productive and non-productive types), ^b^Matlab Health and Demographic Surveillance System 1982–2014, any brother.

**Fig 1:**
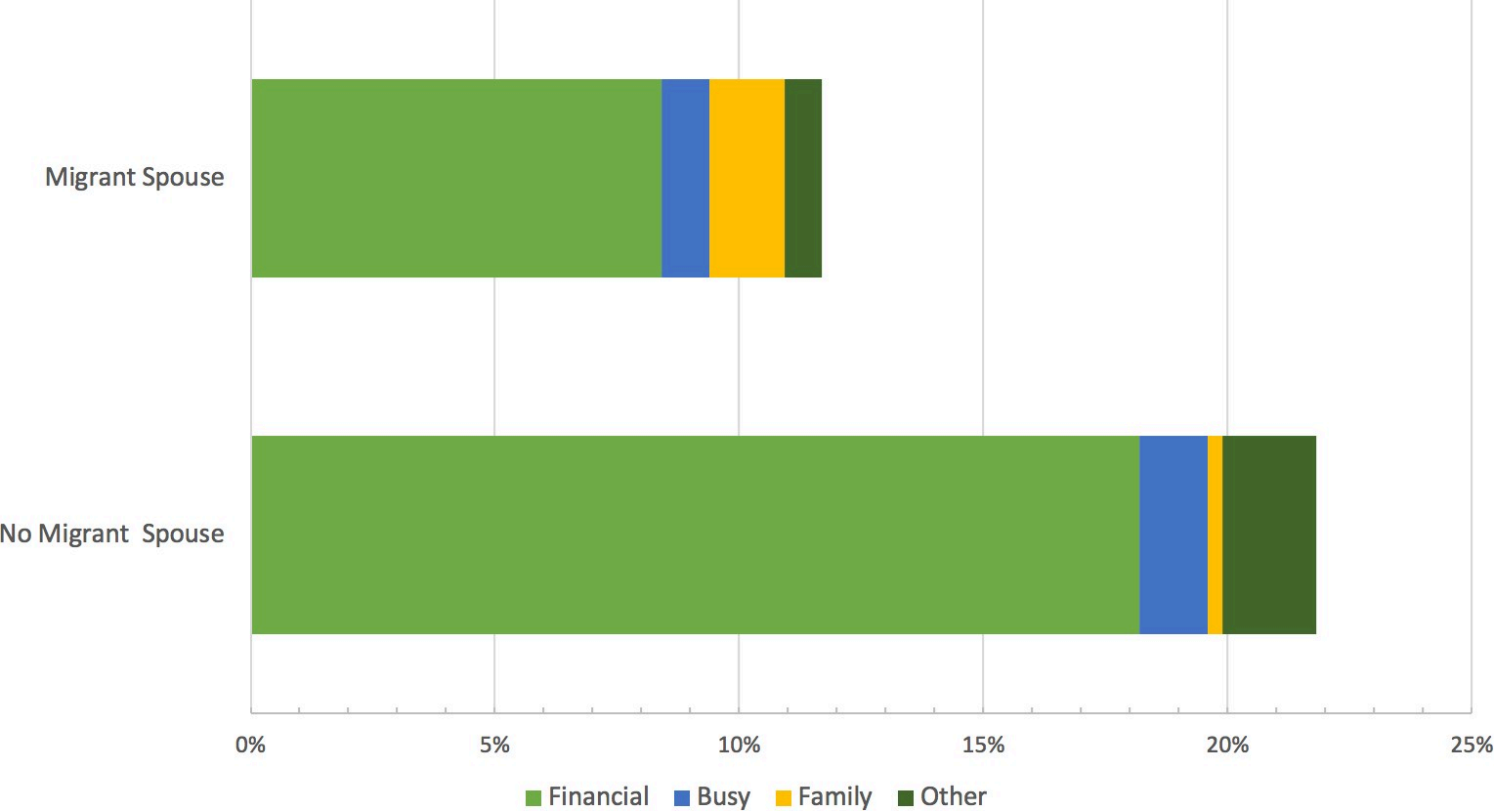
Predicted probability for barriers that prevent access to needed health services for married women. Source: MHSS1 (1996–1997), MHSS2 (2012–2014), Matlab Health & Demographic Surveillance System (1982–2014). Logistic regression predicted probabilities. Other: transportation, too ill, did not know where to go (all <1% of responses) and "other specify". All models control for respondent’s age, education, minor children, relationship to household head, self-reported health, father and siblings’ migration status, household assets, urban vs. rural location, and father’s education.

Fig 1 illustrates that having an international migrant spouse significantly reduced the predicted probability of experiencing any barriers to healthcare utilization for women left behind, primarily through a reduction in financial related barriers. The predicted probability of experiencing a financial related barrier that prevented a woman from accessing needed healthcare was 8.4% for women with international migrant spouses and 18.2% for women without international migrant spouses. The probabilities for family barriers were very small overall, but the predicted probability for women without migrant spouses was more than five times higher, 1.6% compared to 0.3%.

Final multinomial and logistic models for the specific barriers to utilization included controls for both remittances and social contact between spouses. Remittances significantly reduced the probability of experiencing financial barriers to care (coefficient: -0.598, p<0.05). Everyday social contact between spouses reduced financial barriers (coefficient: -1.027, p<0.01) and increased barriers related to being too busy (too much work) (coefficient: 1.820, p<0.05) (Table 4).

Women who lived in multigenerational households and whose household head was a member of their natal/biological family saw statistically significant increases in the probability of experiencing barriers related to being too busy/not getting time off work compared to women who lived in nuclear families (coefficient: 1.740, p<0.05). For women in multigenerational households whose household head was an in-law, we saw increases in other barriers to care (coefficient: 0.911, p<0.05). Living in an urban area significantly reduced financial (coefficient: -1.170, p<0.001) and other (coefficient: -2.482, p<0.001) barriers to care. “Other” barriers include distance, transportation, too ill to travel and other individual reasons.

#### Health status

Self-reported health status was not significantly different between women with and without international migrant spouses in bivariate analyses or multivariate analysis (Appendix E in S1 Appendix). Migration of a spouse was not significantly associated with self-reported health, and other migration related measures including having an emigrant brother or father, remittances, social contact, and household assets were similarly unrelated to self-reported health status in one of the binary specifications. In the specification that compared women with poor or fair health to those with good health, having a father as a migrant and everyday spousal contact were good for health. Conversely, living in an urban area was significantly associated with better self-reported health. In multivariate logistic models of the probability of having poor health, living in an urban area had a coefficient of -1.377, p<0.001. Health status was, however, a significant predictor for the ability to utilize needed health care. Women whose self-reported health was poor were more likely to experience barriers that prevented them from accessing care compared to women whose self-reported health was fairly healthy or healthy (coefficient: 1.650, p<0.001) (Table 3 Model 3). The effect of health status was consistent and significant across all health access models including multinomial models for financial and family related barriers (Table 4).

## Discussion

Our results indicate that migration can have a positive effect on healthcare access and utilization for left-behind women. In this study we observed that wives of international migrants had significantly lower risk of lacking needed healthcare compared to women whose spouses remained in Bangladesh. Because we are able to control for selection into migration using propensity score approaches, we are more confident that migration effects can be attributed to the migration and not to other factors such as wealth, education and the cumulative benefits of past migration. The availability of longitudinal data including extensive family migration histories allowed us to control for the propensity to have a migrant spouse, thereby isolating the effect. Propensity adjustments did little to alter our findings. This is unsurprising in light of an earlier study using the same data that found no propensity effects due to migration on selection on men’s own health, and given the widespread and readily available practice of international migration in the study population [59]. The evidence of spousal migrations’ positive impact on the healthcare utilization for wives in Bangladesh contributes to the healthcare access literature by directing us to look beyond individual factors and toward broader family and community characteristics when seeking to understand the role of enabling, predisposing, and need factors in different contexts [23, 24]. The relationship between migration and health extends beyond the migrants themselves, and the improvements in access for wives of migrants highlights the importance of relational factors in these relationships [61, 62].

One relational factor that we examine is social contact between spouses. Everyday social contact with their husbands had a positive effect on women’s access to care, reducing the probability that they would lack needed healthcare. The effect of everyday contact was similar in the analysis of the full sample of women and the subset that included only women whose spouses did not live with them, illustrating the key role that men can play in facilitating access to care for their wives. Social contact between spouses had different effects on different barriers to healthcare utilization; reducing the probability of experiencing financial barriers and increasing the probability of experiencing barriers related to being too busy and having too many responsibilities. These findings align with literature on transnational families and communication showing the complex gendered influence that men can exert from abroad [44, 63].

The most common barrier to accessing healthcare for all women was financial, and the reduction in barriers overall for wives of international migrants was due in large part to a reduction in these financial barriers to care. This result held across the logistic and multinomial specifications with and without the mediating mechanisms of social contact and remittances. This finding is in line with studies on common barriers to care in a range of settings where healthcare costs lead to deferral and/or delay of care [64–66]. This result may be predictable given the substantial financial returns to overseas migration in a population with well-established migration routes and deep stocks of social capital. Reported annual remittances received from abroad in this sample (mean $2515.53) were quite large in comparison to the price of regular healthcare in Bangladesh, even with over 50% of healthcare costs coming from out-of-pocket payments [67–69], and with evidence showing that remittances are often used for basic consumption [70]. Our results are thus in line with existing evidence and provide insight into some of the specific positive impacts of migration in sending communities. However, our analysis of the specific effects of remittances showed that neither receipt of remittances nor the amount of remittances received were significant predictors of overall healthcare utilization for women in models, but were associated with a reduction in financial barriers in multinomial models. As mentioned in the limitations section below, the temporal differences in the measurement of remittances (1 year) and healthcare utilization (3 years) may mask a direct association between the variables. Remittances received in prior years could be used for a current healthcare need or the practice of borrowing against future remittances could also reduce measured remittances. The impact of remittances on women left behind is complicated and as other studies have found, the benefits are not necessarily always attributable to specific remittance reports or amounts [28].

Our secondary analyses on the barriers women faced in accessing care warrant a more nuanced interpretation and point to scenarios in which financial benefits might not outweigh social costs. We observed significant increases in the probabilities of experiencing family related and other barriers for women with migrant spouses. Results indicate that the potential benefits of migration for women left behind may be diluted by family structures that perpetuate unequal gender dynamics and that women face barriers that cannot be directly addressed through increased financial resources [71]. Household composition matters in whether the effects of spousal migration for women encompass increased autonomy alongside increased responsibility and financial resources [34, 72, 73]. As a result of the migration, women have the potential to face both increasing family barriers and decreasing financial barriers to healthcare utilization [74]. While financial benefits outweighed social costs in this study of a well-developed, relatively lucrative migration stream characterized by high levels of spousal contact, the results might differ in less advantageous settings [75].

### Limitations

Our study design has some inherent limitations. While our data allow for generational controls, and use of methods such as propensity score matching account for treatment selection and improve the reliability of our estimates, we are unable to make causal claims about the relationship between migration and healthcare utilization. Specifically, we do not account for the duration of the spousal migration or the timing of migration in relation to specific episodes of inadequate access to healthcare. Temporal concerns also play a role in the relationship between remittances, which were measured over a 12-month recall, and healthcare utilization measured over a 3-year period. Another limitation is the use of multinomial logistic regression for the measure of distribution of different barriers to healthcare utilization. Although we have a large sample size, so these results are not underpowered, and these models do not assume normality, linearity, or homoscedasticity, we are not able to fully confirm that our data meet the assumption of independence among the dependent variable choices. It is theoretically plausible that membership in one category is related to membership of another category, for example, having financial barriers to healthcare utilization could also lead someone to have to work long hours and therefore they face two barriers (busy and money). To account for this, we ran individual logistic models for dummy variables of each of the multinomial outcomes and the results did not differ significantly from the multinomial models.

Finally, our results did not explore the consequences of the relationship between spousal migration and health care access such as self-reported health or morbidity, and we were not able to look at type or quality of care [26]. While we did some preliminary analyses on the relationship between outcomes such as mental health and women’s empowerment and healthcare utilization for the left-behind, future studies should consider these pathways and include more in-depth analysis of these complex relationships. Our analyses confirm the importance of how families negotiate healthcare in the context of migration, highlighting the direct and indirect pathways through which migration affects the health of women left behind. One of those possible pathways is through women’s empowerment.

## Conclusion

In spite of these limitations, this study raises important policy concerns with respect to barriers and facilitators to health services utilization for women left behind and points to how migration can play a role in the conceptualization of healthcare utilization more broadly. This paper demonstrates that migration can simultaneously play a role in some of the individual pre-disposing and enabling characteristics for health care utilization, and also direct us to the structural and fundamental causes of health and health inequities [24, 76–78]. Sending countries should look to reduce out-of-pocket spending on healthcare, particularly if local wages are not enough to support these costs [67]. To improve access, delivery of services should address not only financial barriers, but also family related barriers that are inherently gendered. In the context of Bangladesh and other low- and middle-income countries, this can take the form of door-step delivery of services or provision of accompaniment services for women. If migration is acting as a form of insurance for healthcare costs, sending countries must address the inequities this presents while also seeking to leverage the benefits of having a significant portion of their working-age population abroad. As posited by the New Economics of Labor Migration framework, migration is a family and community process that implies a very high degree of dependence between migrants and families left behind [79]. The United Nations Sustainable Development Goals and The Global Compact for Safe, Orderly and Regular Migration point to a few policy avenues that recognize this interdependence, including reducing the cost of financial transfers, making a greater proportion of remittances available to left-behind families and improving bilateral cooperation to address the gendered nature of many push and pull migration factors such as those that do not allow for labor migrants to bring their families [80].

This study shows there is a need for further research on the gendered dynamics of migration and the health of those left behind. This analysis of healthcare utilization for women left behind in Bangladesh provides rare evidence on a population that is both understudied and at risk. The comparison of women with and without international migrant spouses, combined with longitudinal data going back many years, allows us to approach an answer to the question of whether spousal migration is good for left-behind families’ healthcare access, and in what ways. Wives of international migrants showed significantly better access to healthcare even when accounting for selection into a migrant family. Yet, in spite of these advantages, wives of migrants may face other challenges in regard to family structure and women’s empowerment.

## Supporting information

S1 Appendix (DOCX)
