# Correction: Effects of spousal migration on access to healthcare for women left behind: A cross-sectional follow-up study

**DOI:** 10.1371/journal.pone.0261872

**Published:** 2021-12-20

**Authors:** The PLOS ONE Staff

## Notice of Republication

An incorrect version of Fig 1 was published in error. This article was republished on December 13, 2021, to correct this. The publisher apologizes for the error. Please download this article again to view the correct version.
