# Supplementary material for: Effects of spousal migration on access to healthcare for women left behind: A cross-sectional follow-up study
Source: PLoS One. 2021 Dec 2;16(12):e0260219. doi: 10.1371/journal.pone.0260219 (PMC8638922; doi:10.1371/journal.pone.0260219)
Supplement: S1 Appendix — (DOCX) [file pone.0260219.s001.docx]

**Supporting Information**

**Appendices A, B, C, D, & E**

Effects of spousal migration on access to healthcare for women left behind:

A cross-sectional follow-up study

Heidi S. West^1*^, Mary E. Robbins^2^, Corrina Moucheraud^1^, Abdur Razzaque^3^, Randall Kuhn^4^

^1^ Department of Health Policy and Management, Fielding School of Public Health, University of California Los Angeles, Los Angeles, CA, United States of America

^2^ Department of Gender Studies, University of California Los Angeles, Los Angeles, CA, United States

of America

^3^ Health and Population Surveillance Division, icddr,b, Dhaka, Bangladesh

^4^ Department of Community Health Sciences, Fielding School of Public Health, University of California

Los Angeles, Los Angeles, CA, United States of America

[*heidiwest15@ucla.edu](mailto:*heidiwest15@ucla.edu) (HSW)

**Appendix A: Sensitivity analyses of spousal migration status across 2 waves of data (MHSS1 1996 and MHSS2 2012-14)**

**Multivariate analysis of characteristics associated with not accessing needed healthcare at M2 (2012-2014)**

|  | 3^+^ | 6 |
| --- | --- | --- |
| Migrant Spouse at M2 (yes/no) | -0.786*** |  |
|  | (0.237) |  |
| ^a^Migrant Spouse over both waves (ref=no migrant spouse) |  |  |
| Migrant Spouse at M1 only |  | -0.603 |
|  |  | (0.566) |
| Migrant Spouse at M2 only |  | -0.747** |
|  |  | (0.243) |
| Migrants Spouse M1 & M2 |  | -1.770* |
|  |  | (0.790) |
| Age (ref=15-19 years) |  |  |
| 20-24 | 0.272 | 0.269 |
|  | (0.480) | (0.480) |
| 25-29 | 0.415 | 0.411 |
|  | (0.461) | (0.461) |
| 30-34 | 0.761 | 0.755 |
|  | (0.468) | (0.469) |
| 35-39 | 0.740 | 0.772 |
|  | (0.475) | (0.476) |
| 40-44 | 0.745 | 0.778 |
|  | (0.471) | (0.472) |
| Respondent's education (ref = none) |  |  |
| 1-4 years | -0.0429 | -0.0376 |
|  | (0.224) | (0.224) |
| 5-9 years | -0.323 | -0.324 |
|  | (0.237) | (0.239) |
| 10+ years | -1.394*** | -1.411*** |
|  | (0.321) | (0.322) |
| Has minor children at home | 0.684* | 0.675* |
|  | (0.272) | (0.276) |
| Self-reported health (ref=healthy) |  |  |
| Fairly healthy | 0.933*** | 0.938*** |
|  | (0.146) | (0.147) |
| Unhealthy/poor health | 1.650*** | 1.646*** |
|  | (0.196) | (0.196) |
| Family Structure (ref: nuclear - head/wife of head) |  |  |
| Multigen: Head is Bio/Natal | 0.210 | 0.203 |
|  | (0.324) | (0.323) |
| Multigen: Head is In-Law | 0.0552 | 0.0615 |
|  | (0.198) | (0.199) |
| Lives in urban area | -1.163*** | -1.161*** |
|  | (0.209) | (0.209) |
| ^b^Household Assets (log) | -0.152** | -0.146** |
|  | (0.0534) | (0.0543) |
| Father's Education (ref= none) |  |  |
| 1-4 years | -0.0459 | -0.0527 |
|  | (0.202) | (0.202) |
| 5-9 years | -0.191 | -0.200 |
|  | (0.183) | (0.184) |
| 10+ years | 0.0285 | 0.0345 |
|  | (0.246) | (0.249) |
| ^c^Father was international migrant | 0.506 | 0.496 |
|  | (0.303) | (0.303) |
| ^c^Brother was international migrant | -0.140 | -0.132 |
|  | (0.164) | (0.164) |
| Observations | 3,187 | 3,187 |

Source: MHSS2 (2012–2014) except where noted, *** p<0.001, ** p<0.01, * p<0.05, Logistic Regression coefficients with robust standard errors in parentheses. ^+^ Model 3=Model 3 from Table 3: primary regressor based on M2 spousal migration status only

^a^Women not married at M1 are categorized based on status of their spouse at M2 "Migrant Spouse at M2 only" or” No migrant spouse"

^b^ MHSS1 1996-1997 for wife’s household (sum value of assets across all productive and non-productive types)

^c^ Matlab Health and Demographic Surveillance System 1982–2014, any brother

**Appendix B Different specification of remittances (amount)**

**Multivariate analysis of characteristics associated with being prevented from accessing needed healthcare**

|  | 1 | 2 |
| --- | --- | --- |
| Has International Migrant Spouse | -0.467 | -0.625* |
|  | (0.293) | (0.315) |
| Age (ref=15-19 years) |  |  |
| 20-24 | 0.281 | 0.290 |
|  | (0.473) | (0.480) |
| 25-29 | 0.417 | 0.446 |
|  | (0.454) | (0.463) |
| 30-34 | 0.791 | 0.843 |
|  | (0.460) | (0.468) |
| 35-39 | 0.744 | 0.779 |
|  | (0.469) | (0.477) |
| 40-44 | 0.746 | 0.797 |
|  | (0.466) | (0.475) |
| Respondent's education (ref = none) |  |  |
| 1-4 years | -0.0324 | -0.0328 |
|  | (0.224) | (0.224) |
| 5-9 years | -0.295 | -0.274 |
|  | (0.238) | (0.237) |
| 10+ years | -1.369*** | -1.343*** |
|  | (0.321) | (0.321) |
| Has minor children at home | 0.681* | 0.686* |
|  | (0.272) | (0.272) |
| Self-reported health (ref=healthy) |  |  |
| Fairly healthy | 0.935*** | 0.927*** |
|  | (0.147) | (0.146) |
| Unhealthy/poor health | 1.642*** | 1.648*** |
|  | (0.198) | (0.200) |
| Family Structure (ref: nuclear- head/wife of head) |  |  |
| Multigen: Head is Bio/Natal | 0.229 | 0.175 |
|  | (0.321) | (0.335) |
| Multigen: Head is In-Law | 0.0372 | 0.0273 |
|  | (0.200) | (0.199) |
| Lives in urban area | -1.180*** | -1.170*** |
|  | (0.211) | (0.211) |
| ^a^Household Assets (log) | -0.148** | -0.145** |
|  | (0.0533) | (0.0532) |
| Father's Education (ref= none) |  |  |
| 1-4 years | -0.0559 | -0.0606 |
|  | (0.204) | (0.205) |
| 5-9 years | -0.195 | -0.195 |
|  | (0.183) | (0.183) |
| 10+ years | 0.0292 | 0.0482 |
|  | (0.246) | (0.248) |
| ^b^Father was international migrant | 0.487 | 0.505 |
|  | (0.304) | (0.308) |
| ^b^Brother was international migrant | -0.152 | -0.159 |
|  | (0.165) | (0.165) |
| Remittances Amount (sqrt) | -0.00134 | -0.00165 |
|  | (0.000842) | (0.000873) |
| Everyday contact or spouse is co-resident |  | -0.587* |
|  |  | (0.276) |
| Observations | 3,187 | 3,187 |

Source: MHSS2 (2012–2014) except where noted, *** p<0.001, ** p<0.01, * p<0.05, Logistic Regression coefficients with robust standard

errors in parentheses. ^a^ MHSS1 1996-1997 for wife’s household (sum value of assets across all productive and non-productive types), ^b^Matlab Health and Demographic Surveillance System 1982–2014, any brother

**Appendix C: Social Contact**

**Multivariate analysis of social contact and access to needed health care for women with non-coresident spouses only**

|  | 1 | 2 | 3 |
| --- | --- | --- | --- |
| Has International Migrant Spouse | -0.819** | -0.769* | -0.768* |
|  | (0.301) | (0.317) | (0.317) |
| Everyday contact with spouse | -0.712* | -0.684* | -0.678* |
|  | (0.284) | (0.289) | (0.290) |
| Age (ref=15-19 years) |  |  |  |
| 20-24 | 0.107 | 0.142 | 0.159 |
|  | (0.787) | (0.856) | (0.871) |
| 25-29 | 0.380 | 0.218 | 0.227 |
|  | (0.807) | (0.834) | (0.843) |
| 30-34 | 0.379 | 0.374 | 0.387 |
|  | (0.864) | (0.860) | (0.875) |
| 35-39 | 0.780 | 0.565 | 0.584 |
|  | (0.873) | (0.883) | (0.900) |
| 40-44 | 0.886 | 0.459 | 0.469 |
|  | (0.868) | (0.934) | (0.945) |
| Respondent's edu (ref = none) |  |  |  |
| 1-4 years | -0.0841 | -0.0916 | -0.0871 |
|  | (0.517) | (0.561) | (0.559) |
| 5-9 years | -0.316 | -0.613 | -0.595 |
|  | (0.442) | (0.528) | (0.528) |
| 10+ years | -1.296* | -1.525* | -1.508* |
|  | (0.569) | (0.700) | (0.696) |
| Has minor children at home | 0.318 | 0.495 | 0.513 |
|  | (0.423) | (0.450) | (0.435) |
| Self-reported health (ref=healthy) |  |  |  |
| Fairly healthy |  | 1.133*** | 1.130*** |
|  |  | (0.294) | (0.294) |
| Unhealthy/poor health |  | 2.237*** | 2.221*** |
|  |  | (0.426) | (0.432) |
| Family Structure (ref: nuclear- head/wife of head) |  |  |  |
| Multigen: Head is Bio/Natal |  | 0.453 | 0.438 |
|  |  | (0.407) | (0.400) |
| Multigen: Head is In-Law |  | 0.293 | 0.286 |
|  |  | (0.342) | (0.344) |
| Lives in urban area |  | -0.532 | -0.537 |
|  |  | (0.622) | (0.622) |
| ^a^Household Assets (log) |  | -0.188 | -0.187 |
|  |  | (0.0991) | (0.0999) |
| Father's Education (ref= none) |  |  |  |
| 1-4 years |  | 0.408 | 0.393 |
|  |  | (0.429) | (0.433) |
| 5-9 years |  | 0.559 | 0.561 |
|  |  | (0.350) | (0.351) |
| 10+ years |  | 0.575 | 0.574 |
|  |  | (0.466) | (0.463) |
| ^b^Father was international migrant |  | 0.0612 | 0.0424 |
|  |  | (0.560) | (0.574) |
| ^b^Brother was international migrant |  | -0.335 | -0.358 |
|  |  | (0.321) | (0.324) |
| Received Remittances |  |  | -0.178 |
|  |  |  | (0.424) |
| Observations | 938 | 938 | 938 |

Source: MHSS2 (2012–2014) except where noted, *** p<0.001, ** p<0.01, * p<0.05, Logistic Regression coefficients with robust standard errors in parentheses. ^a^MHSS1 1996-1997 for wife’s household (sum value of assets across all productive and non-productive types). ^b^ Matlab Health and Demographic Surveillance System 1982–2014, any brother

**Appendix D Propensity Models: Multivariate analysis of characteristics associated with being prevented from accessing needed healthcare with treatment selection controls**

|  | 1 | 2 | 3 | 4 |
| --- | --- | --- | --- | --- |
|  | ACS | ACS + Prop. Block | ACS + Prop Score | ACS + PS + Remit |
| Has International Migrant Spouse | -0.768*** | -0.768** | -0.780** | -0.766** |
|  | (0.229) | (0.235) | (0.237) | (0.290) |
| Age (ref=15-19 years) |  |  |  |  |
| 20-24 | 0.256 | 0.353 | 0.250 | 0.264 |
|  | (0.494) | (0.490) | (0.495) | (0.506) |
| 25-29 | 0.375 | 0.452 | 0.365 | 0.393 |
|  | (0.481) | (0.476) | (0.485) | (0.498) |
| 30-34 | 0.686 | 0.776 | 0.677 | 0.723 |
|  | (0.491) | (0.486) | (0.496) | (0.510) |
| 35-39 | 0.687 | 0.723 | 0.670 | 0.690 |
|  | (0.499) | (0.509) | (0.508) | (0.521) |
| 40-44 | 0.715 | 0.718 | 0.726 | 0.757 |
|  | (0.496) | (0.488) | (0.492) | (0.506) |
| Respondent's education (ref = none) |  |  |  |  |
| 1-4 years | -0.0686 | 0.0346 | -0.0815 | -0.0749 |
|  | (0.224) | (0.267) | (0.230) | (0.230) |
| 5-9 years | -0.332 | -0.207 | -0.374 | -0.326 |
|  | (0.235) | (0.329) | (0.270) | (0.271) |
| 10+ years | -1.392*** | -1.219** | -1.439*** | -1.390*** |
|  | (0.315) | (0.404) | (0.357) | (0.358) |
| Has minor children at home | 0.698** | 0.708* | 0.702** | 0.692* |
|  | (0.270) | (0.276) | (0.272) | (0.275) |
| Poor Self Reported Health | 0.821*** | 0.840*** | 0.822*** | 0.819*** |
|  | (0.0929) | (0.0929) | (0.0927) | (0.0937) |
| Family Structure (ref: nuclear- head/wife of head) | |  |  |  |
| Multigen: Head is Bio/Natal | 0.221 | 0.0656 | 0.103 | 0.183 |
|  | (0.320) | (0.471) | (0.504) | (0.513) |
| Multigen: Head is In-Law | 0.0808 | 0.0947 | 0.0196 | 0.0458 |
|  | (0.199) | (0.304) | (0.296) | (0.294) |
| Lives in urban area | -1.118*** | -1.213*** | -1.080*** | -1.099*** |
|  | (0.211) | (0.267) | (0.231) | (0.234) |
| ^a^Household Assets (log) | -0.160** | -0.144* | -0.169** | -0.166** |
|  | (0.0542) | (0.0617) | (0.0571) | (0.0572) |
| Father's Education (ref= none) |  |  |  |  |
| 1-4 years | -0.0271 | -0.0641 | -0.0257 | -0.0411 |
|  | (0.201) | (0.202) | (0.201) | (0.203) |
| 5-9 years | -0.187 | -0.212 | -0.185 | -0.189 |
|  | (0.183) | (0.184) | (0.184) | (0.183) |
| 10+ years | 0.0587 | -0.0381 | 0.0579 | 0.0840 |
|  | (0.250) | (0.256) | (0.250) | (0.251) |
| Household Received Remittances |  |  |  | -0.350 |
|  |  |  |  | (0.209) |
| Everyday contact or spouse is co-resident |  |  |  | -0.634* |
|  |  |  |  | (0.286) |
| Estimated propensity score |  |  | 0.446 | 0.354 |
|  |  |  | (1.397) | (1.415) |
| Propensity Block |  |  |  |  |
| 1 |  | 0.0769 |  |  |
|  |  | (1.091) |  |  |
| 2 |  | -0.176 |  |  |
|  |  | (0.993) |  |  |
| 3 |  | -0.143 |  |  |
|  |  | (0.954) |  |  |
| 4 |  | -0.0167 |  |  |
|  |  | (0.930) |  |  |
| 5 |  | -0.306 |  |  |
|  |  | (0.937) |  |  |
| 6 |  | -0.811 |  |  |
|  |  | (0.906) |  |  |
| 7 |  | 0.274 |  |  |
|  |  | (0.872) |  |  |
| 8 |  | -0.126 |  |  |
|  |  | (0.877) |  |  |
| Observations | 3,180 | 3,180 | 3,180 | 3,180 |

Source: MHSS2 (2012–2014) except where noted

Logistic Regression coefficients with robust standard errors in parentheses

*** p<0.001, ** p<0.01, * p<0.05

^a^ MHSS1 1996-1997 for wife’s household (sum value of assets across all productive and non-productive types)

**Appendix E: Self-Reported Health Outcome Models**

**Multivariate analysis of characteristics associated with poor self-reported health**

|  | 1 Poor Health | 2. Poor Health | 3. Poor/Fair Health | 4. Poor/Fair Health |
| --- | --- | --- | --- | --- |
| Has International Migrant Spouse | -0.259 | 0.0508 | -0.113 | -0.0890 |
|  | (0.263) | (0.362) | (0.140) | (0.180) |
| Age (ref=15-19 years) |  |  |  |  |
| 20-24 | 0.576 | 0.592 | 0.332 | 0.319 |
|  | (0.521) | (0.521) | (0.268) | (0.268) |
| 25-29 | 1.061* | 1.070* | 0.694** | 0.689** |
|  | (0.520) | (0.521) | (0.265) | (0.264) |
| 30-34 | 1.033 | 1.053 | 0.754** | 0.760** |
|  | (0.547) | (0.547) | (0.276) | (0.276) |
| 35-39 | 1.493** | 1.496** | 1.400*** | 1.390*** |
|  | (0.548) | (0.548) | (0.283) | (0.283) |
| 40-44 | 1.311* | 1.307* | 1.359*** | 1.358*** |
|  | (0.546) | (0.546) | (0.291) | (0.291) |
| Respondent's education (ref = none) | |  |  |  |
| 1-4 years | 0.383 | 0.391 | 0.0775 | 0.0791 |
|  | (0.298) | (0.299) | (0.198) | (0.198) |
| 5-9 years | 0.464 | 0.489 | 0.261 | 0.283 |
|  | (0.284) | (0.286) | (0.194) | (0.195) |
| 10+ years | 0.135 | 0.153 | 0.0336 | 0.0619 |
|  | (0.384) | (0.383) | (0.228) | (0.229) |
| Has minor children at home | -0.502 | -0.519 | -0.0974 | -0.102 |
|  | (0.270) | (0.270) | (0.180) | (0.180) |
| Family Structure (ref: nuclear- head/wife of head) | |  |  |  |
| Multigen: Head is Bio/Natal | 0.144 | 0.273 | -0.0401 | -0.00148 |
|  | (0.409) | (0.412) | (0.260) | (0.259) |
| Multigen: Head is In-Law | -0.104 | -0.0814 | 0.182 | 0.184 |
|  | (0.243) | (0.245) | (0.142) | (0.142) |
| Lives in urban area | -1.377*** | -1.404*** | -0.519*** | -0.525*** |
|  | (0.254) | (0.253) | (0.135) | (0.135) |
| ^a^Household Assets (log) | 0.0755 | 0.0753 | 0.0790* | 0.0820* |
|  | (0.0567) | (0.0571) | (0.0394) | (0.0396) |
| Father's Education (ref= none) |  |  |  |  |
| 1-4 years | 0.0729 | 0.0615 | 0.106 | 0.0922 |
|  | (0.235) | (0.235) | (0.150) | (0.150) |
| 5-9 years | -0.156 | -0.163 | 0.0503 | 0.0493 |
|  | (0.207) | (0.207) | (0.132) | (0.132) |
| 10+ years | -0.596* | -0.587* | -0.364* | -0.349 |
|  | (0.294) | (0.293) | (0.186) | (0.186) |
| ^b^Father was international migrant | -0.595 | -0.588 | -0.665** | -0.644** |
|  | (0.372) | (0.368) | (0.206) | (0.206) |
| ^b^Brother was international migrant | 0.0499 | 0.0416 | 0.0125 | 0.00717 |
|  | (0.187) | (0.186) | (0.117) | (0.117) |
| Received Remittances |  | -0.417 |  | -0.205 |
|  |  | (0.282) |  | (0.159) |
| Everyday contact or spouse is co-resident |  | -0.0155 |  | -0.414* |
|  |  | (0.327) |  | (0.192) |
| Observations | 3,187 | 3,187 | 3,187 | 3,187 |

Source: MHSS2 (2012–2014) except where noted

Logistic Regression coefficients with robust standard errors in parentheses

*** p<0.001, ** p<0.01, * p<0.05

^a^ MHSS1 1996-1997 for wife’s household (sum value of assets across all productive and non-productive types)

^b^  Matlab Health and Demographic Surveillance System 1982–2014, any brother
